# Homogeneously *N*-glycosylated proteins derived from the GlycoDelete HEK293 cell line enable diffraction-quality crystallogenesis

**DOI:** 10.1107/S2059798320013753

**Published:** 2020-11-24

**Authors:** Sandra Kozak, Yehudi Bloch, Steven De Munck, Aleksandra Mikula, Isabel Bento, Savvas N. Savvides, Rob Meijers

**Affiliations:** a European Molecular Biology Laboratory (EMBL), Hamburg Outstation, Notkestrasse 85, 22607 Hamburg, Germany; b Unit for Structural Biology, Department of Biochemistry and Microbiology, Ghent University, Technologiepark-Zwijnaarde 71, 9052 Ghent, Belgium; c Unit for Structural Biology, VIB Center for Inflammation Research, Technologiepark-Zwijnaarde 71, 9052 Ghent, Belgium; d Institute for Protein Innovation, 4 Blackfan Circle, Boston, MA 02115, USA

**Keywords:** glycoproteins, synthetic biology, crystallization, cell-surface receptors, glycosylation, GlycoDelete cell line

## Abstract

Structural studies of glycoproteins and their complexes provide critical insights into their roles in normal physiology and disease. Most glycoproteins contain N-linked glycosylation, a key post-translation modification that critically affects protein folding and stability and the binding kinetics underlying protein interactions. However, N-linked glycosylation is often an impediment to yielding homogeneous protein preparations for structure determination by X-ray crystallography or other methods. In particular, obtaining diffraction-quality crystals of such proteins and their complexes often requires modification of both the type of glycosylation patterns and their extent. Here, we demonstrate the benefits of producing target glycoproteins in the GlycoDelete human embryonic kidney 293 cell line that has been engineered to produce N-glycans as short glycan stumps comprising *N*-acetylglucosamine, galactose and sialic acid. Protein fragments of human Down syndrome cell-adhesion molecule and colony-stimulating factor 1 receptor were obtained from the GlycoDelete cell line for crystallization. The ensuing reduction in the extent and complexity of *N*-glycosylation in both protein molecules compared with alternative glyco­engineering approaches enabled their productive deployment in structural studies by X-ray crystallography. Furthermore, a third successful implementation of the GlycoDelete technology focusing on murine IL-12B is shown to lead to *N*-glycosylation featuring an immature glycan in diffraction-quality crystals. It is proposed that the GlycoDelete cell line could serve as a valuable go-to option for the production of homogeneous glycoproteins and their complexes for structural studies by X-ray crystallography and cryo-electron microscopy.

## Abstract 2

Structural studies of glycoproteins are often complicated by glycan complexity. Here, it is shown that the GlycoDelete HEK293 cell line engineered to produce glycan stumps produces homogeneous glycoproteins that are ideal for crystallogenesis and other structural studies.

## 1. Introduction   

*N*-Glycosylation (NG) is a prominent protein post-translational modification that involves the covalent linkage of a carbo­hydrate to an asparagine. NG sites can be predicted on the basis of the amino-acid sequence surrounding the affected amino acid, based on the consensus sequence Asn-*X*-Thr/Ser, where *X* is any amino acid except for proline (Apweiler *et al.*, 1999 ▸). *N*-Glycosylation is encountered in more than 10% of the mammalian proteome (Zielinska *et al.*, 2010 ▸) and is enriched in proteins following the secretory pathway, such as cell-surface receptors, cell-adhesion molecules and secreted glycoproteins (Zielinska *et al.*, 2012 ▸). To employ *N*-glycosyl­ated proteins in structural studies, it is often imperative to ensure proper glycosylation during protein expression. Since bacterial systems do not offer a comparable glycosylation machinery, several eukaryotic protein-expression systems are specifically used to ensure proper *N*-glycosylation. The most commonly used expression systems for the production of recombinant glycoproteins are human embryonic kidney 293 (HEK293), Chinese hamster ovary (CHO), *Spodoptera frugiperda* (*e.g.* Sf9), *Trichoplusia ni* (*e.g.* HighFive), *Dros­ophila melanogaster* (*e.g.* S2) and *Pichia pastoris* cells.

The chemical composition of N-glycans differs among species and may impact protein folding and expression yield when heterologous proteins are expressed (Chang *et al.*, 2007 ▸). Assembled from a common pentasaccharide core, mammalian *N*-glycosylation is complex and the ensuing glycan trees display a wide variety of branches of varying sugar composition. Although mammalian protein-expression systems are relatively costly, they are preferred for the expression of secreted mammalian proteins to ensure native-like glycosyl­ation, which should support protein folding and function. However, crystallogenesis can be hampered by the extent and heterogeneity of N-glycans. This is because such post-translational modification sites are found on the protein surface and may interfere with potential crystal-packing interfaces that are necessary to construct a well ordered crystalline lattice. Larger, branched glycan structures may introduce conformational flexibility; while they can facilitate crystallization, the diffraction properties of these crystals are often poor.

Here, we present the use of the GlycoDelete (GD) HEK cell line for protein crystallization, which produces homogeneous mature-like trisaccharide glycan stubs (Meuris *et al.*, 2014 ▸). The glycan stubs do not need further enzymatic processing, and we show that for heavily glycosylated fragments of human colony-stimulating factor 1 receptor (CSF-1R), murine IL-12B and human Down syndrome cell-adhesion molecule (DSCAM) the NG modifications facilitate the production of diffraction-quality crystals. The GD cell line is derived from the HEK293S *MGAT1^−/−^* cell line and has been transformed with Golgi-resident EndoT (an endo-β-*N*-acetyl­glucosaminidase similar to EndoH). This enzyme processes the Man_5_GlcNAc_2_ glycans, allowing the NG to be further processed by native Golgi-resident enzymes to generate mature-like glycans consisting of one GlcNac (which can be fucosylated), one galactose (Gal) and one sialic acid (Neu_5_Ac). We present a detailed analysis of the glycan structures of the crystallized proteins and show examples where the use of the GD cell line improves crystal packing and alters dynamics in glycan maturation. Together, these examples suggest that the GD cell line can serve as a key option to obtain homogeneous samples of glycoproteins for structural studies, in particular for the improvement of the X-ray diffraction properties of *N*-glycosylated proteins.

## 2. Materials and methods   

### 2.1. Cloning and expression of receptor fragments   

To facilitate crystallization of the netrin-binding region of human DSCAM_Ig7–Ig9_, the DSCAM fragment covering residues 595–884 was codon-optimized and synthesized by GenScript based on the amino-acid sequence from UniProt ID O60469. The fragment was inserted into the pXLG vector (Backliwal *et al.*, 2008 ▸) using restriction-site cloning between a 34-residue signal peptide from pregnancy-specific glyco­protein 1 (UniProt ID P11464) and a 6×His C-terminal tag followed by two stop codons (Finci *et al.*, 2014 ▸).

The HEK293 GlycoDelete cell line was obtained at passage number 10 from a stock kindly provided by Nico Callewaert (VIB–UGent Center for Medical Biotechnology, Ghent, Belgium), and cells from this passage number were cultured for adaptation to adherent conditions. The cells from subsequent passage numbers were used for protein expression until a maximum passage number of 20. For large-scale expression, both wild-type HEK293T (ATCC CRL-11268) and HEK293 GD cells were seeded into roller bottles. The GD cells are less adherent, and required a low roller speed incubation period of 4 h following transfer. After 72 h, the cells were transfected with DNA–polyethylenimine (PEI) complexes. Per roller bottle (300 ml expression volume), 50 ml transfection mixture containing 500 µg DNA at a 1:1 DNA:PEI ratio in Dulbecco’s Modified Eagle’s Medium (DMEM) was used. The GD cells were maintained at higher serum concentrations (normally 10%; 1% during expression) than wild-type cells (normally 2%; 1–0.1% during expression). The conditioned media were harvested five days after transfection, filtered (0.27 µm) and supplemented with 1 m*M* sodium azide to prevent microbial growth. Protein yields from a 2.4 l GD culture were typically 0.7 mg l^−1^ after nickel-affinity purification, size-exclusion chromatography (SEC) and sample concentration, whereas the wild-type cells yielded 1.25 mg l^−1^. This reduction in protein yield from the engineered cell line is similar to that observed for HEK293S *MGAT1^−/−^* cells.

For murine IL-12B, the reference sequence (NM_001303244.1) encoding the full-length protein (residues Met1–Ser335) was synthesized by GeneArt and was a kind gift from Complix n.v., Ghent, Belgium. The coding sequence was cloned between the EcoRI and KpnI sites in the pHLsec vector in frame with a C-terminal hexahistidine tag. Protein expression was performed in accordance with previously published protocols (Aricescu *et al.*, 2006 ▸). In brief, adherent GD cells growing in DMEM + 10% fetal calf serum (FCS) were expanded in T175 cell-culture-treated flasks under standard sterile cell-culture techniques. Upon reaching 80% confluency, the medium was exchanged for serum-free DMEM supplemented with 3.6 m*M* valproic acid (VPA) and the cells were transfected utilizing branched 25 kDa PEI as a transfection reagent. A 1:1.5 plasmid:PEI ratio was used. The conditioned medium was harvested five days post-transfection and was clarified by centrifugation and filtration prior to purification. The C-terminal 6×His tag enabled capture of the protein from the conditioned medium by immobilized metal-affinity chromatography (IMAC) followed by size-exclusion chromatography (SEC). The size-exclusion chromatogram clearly revealed both monomeric and dimeric IL-12B. The dimeric fractions were pooled and finally polished by anion exchange on Mono Q resin. The loading and elution buffers for anion exchange were low-salt HBS (20 m*M* HEPES pH 7.4, 50 m*M* NaCl) and high-salt HBS (20 m*M* HEPES pH 7.4, 500 m*M* NaCl), respectively. Elution from the Mono Q column was performed with a linear gradient, in which the IL-12B homodimer eluted at a conductivity of 16 mS cm^−1^.

The cloning, expression and purification of human CSF-1R have previously been reported (Felix *et al.*, 2015 ▸; Elegheert *et al.*, 2011 ▸). In brief, for CSF-1R comprising extracellular domains 1–3 (CSF-1R_D1–D3_), we expressed the glycosylation mutants N73Q, N153Q, N240Q and N275Q in HEK293T and HEK293S *MGAT1^−/−^* cells. All cell lines were grown adherently in 175 cm^3^ tissue-culture flasks in DMEM supplemented with 10% FCS and 3.6 m*M* VPA until they reached 70–80% confluency. The medium was then exchanged to serum-free DMEM supplemented with 3.6 m*M* VPA and the cells were transfected by adding DNA:PEI complexes in a 1:1.5 ratio. The conditioned medium was harvested five days post-transfection, centrifuged for 10 min at 6000*g* and filtered through a 0.22 µm filter. Both cell lines produced about 3 mg of CSF-1R_D1–D3_ per litre of culture after purification by IMAC and SEC.

### 2.2. Crystallization   

Purified DSCAM_Ig7–Ig9_ expressed in both HEK293T and HEK293 GlycoDelete cells was concentrated in 20 m*M* HEPES pH 7.4, 100 m*M* NaCl, 1 m*M* DTT using a 10 kDa Amicon Ultra-4 centrifugal unit to 5 and 7.5 mg ml^−1^, respectively, and subjected to crystallization screening in 100 nl + 100 nl sitting drops using a Mosquito robot (TTP Labtech). DSCAM_Ig7–Ig9_ obtained from wild-type HEK293T cells crystallized within seven days at 19°C in a rod-like morphology in 0.05 *M* magnesium acetate, 10%(*w*/*v*) PEG 8000, 0.1 *M* sodium acetate (condition H9 from The PEGs II Suite, Qiagen). Despite several crystal-optimization attempts, the crystals of HEK293T-expressed DSCAM_Ig7–Ig9_ yielded only poor diffraction (∼8–13 Å). Cube-shaped crystals of DSCAM_Ig7–Ig9_ obtained by HEK293 GlycoDelete expression appeared within 1–3 days in 0.2 *M* calcium acetate, 10%(*w*/*v*) PEG 8000, 0.1 *M* HEPES pH 7.5 (condition H8 from The PEGs II Suite, Qiagen). Further optimization by additive screening (Additive Screen, Hampton Research) and/or manual hanging-drop vapor-diffusion experiments containing a 1.2 µl volume of protein solution and a 1.8 µl volume of mother liquor yielded single and larger crystals that diffracted to resolutions in the range 1.85–3 Å. The best diffracting crystal (1.85 Å resolution) was obtained in 3%(*v*/*v*) glycerol, 0.2 *M* calcium acetate, 10%(*w*/*v*) PEG 8000, 0.1 *M* HEPES pH 7.5. Crystallization conditions are summarized in Table 1 ▸.

For IL-12B, the top elution fraction from anion-exchange chromatography was already found to be highly concentrated at ∼7 mg ml^−1^ and was therefore used in crystallization trials. Extensive commercial sparse-matrix screens were set up with 100 nl protein solution and 100 nl mother liquor in a sitting-drop geometry with a Mosquito robot at 287 K. Multiple hits were identified and optimized. An original hit in condition F2 of the Morpheus I screen from Molecular Dimensions (120 m*M* monosaccharides, 50 m*M* imidazole, 50 m*M* MES pH 6.5, 20% ethylene glycol, 10% PEG 8000) gave rise to the best diffracting crystal.

Crystallization of the CSF-1–CSF-1R_D1–D3_ complex has previously been reported (Felix *et al.*, 2015 ▸). In brief, the top fractions from the final size-exclusion chromatography of complexes of glycosylation mutants of CSF-1R_D1–D3_ produced in HEK293T, HEK293S *MGAT1^−/−^* or GD cells were concentrated to approximately 7.4 mg ml^−1^ and used to set up multiple commercial sparse-matrix screens. Crystallization screens were set up by mixing 100 nl protein solution and 100 nl mother liquor in both sitting-drop and hanging-drop vapor-diffusion geometry using a Mosquito robot (TTP Labtech). The best diffracting crystals were obtained from CSF-1R_D1–D3_ N240Q expressed in HEK293 GD cells in a condition consisting of 0.2 *M* lithium sulfate monohydrate, 0.1 *M* Tris pH 8.5, 28%(*w*/*v*) PEG 3350. The crystals were incubated in stabilizing solution consisting of 35%(*w*/*v*) PEG 3350, 5% ethylene glycol prior to flash-cooling.

### 2.3. Data collection, crystal structure determination and refinement   

Crystals of DSCAM_Ig7–Ig9_ expressed in HEK293 Glyco­Delete cells were harvested and soaked in a cryoprotectant solution consisting of 0.2 *M* calcium acetate, 10%(*w*/*v*) PEG 8000, 0.1 *M* HEPES pH 7.5, 20%(*v*/*v*) glycerol prior to flash-cooling to 100 K. X-ray diffraction data were collected at EMBL Hamburg on the P13 beamline at the PETRA III synchrotron, DESY, Hamburg equipped with a PILATUS 6M detector (25 Hz, 450 µm sensor thickness) and an MD2 diffractometer (Cipriani *et al.*, 2007 ▸). The data were processed using *XDS* (Kabsch, 2010 ▸) and merged and scaled with *AIMLESS* (Evans & Murshudov, 2013 ▸), yielding a data set to 1.85 Å resolution. The crystal belonged to the monoclinic space group *C*2, with unit-cell parameters *a* = 78.6, *b* = 71.4, *c* = 92.2 Å, β = 113.1°. Crystal solvent-content analysis based on the Matthews coefficient indicated the presence of one molecule in the asymmetric unit, corresponding to 67% solvent content. The structure was determined by molecular replacement with *MOLREP* (Vagin & Teplyakov, 2010 ▸) using the structure of the *Drosophila* DSCAM Ig7 domain (PDB entry 4wvr; Li *et al.*, 2016 ▸) as a search model. Two domains, assigned as Ig7 and Ig9, were found by *MOLREP*, and the missing Ig8 domain was built based on structural alignment with the Ig7–Ig8 fragment from the *Drosophila* DSCAM Ig1–Ig8 structure (PDB entry 3dmk; Sawaya *et al.*, 2008 ▸) using the Ig7 domain. The model was refined by ten cycles of rigid-body refinement followed by iterative cycles of restrained refinement and manual model corrections. TLS refinement was conducted in the later stages of the refinement. Refinement steps were performed with *REFMAC*5 (Murshudov *et al.*, 2011 ▸) with a randomly selected 5% of reflections used for cross-validation. Manual model building and the addition of water and N-glycan residues was performed in *Coot* (Emsley *et al.*, 2010 ▸). The model was refined to an *R* factor of 0.189 and an *R*_free_ of 0.217, and its stereochemistry was validated using the *MolProbity* web service (Chen *et al.*, 2010 ▸), indicating that there were no residues in the disallowed regions of the Ramachandran plot. The final data-collection and refinement statistics are summarized in Table 2 ▸.

For IL-12B, the crystals were cryoprotected in mother liquor and vitrified in liquid nitrogen. X-ray diffraction data were collected at EMBL Hamburg on the P14 beamline of the PETRA III synchrotron, DESY, Hamburg equipped with a PILATUS 6M detector and an MD3 diffractometer. The data were integrated and scaled using *XDS* (Kabsch, 2010 ▸), yielding a data set to 2.40 Å resolution. The crystal belonged to the tetragonal space group *I*4_1_ (No. 80), with unit-cell parameters *a* = *b* = 85.88, *c* = 107.67 Å. A high Wilson *B* factor of 61.1 Å^2^ was reported by *XDS*. Solvent-content analysis based on the Matthews coefficient indicated the presence of one molecule in the asymmetric unit with 43% solvent content. The structure was solved by maximum-likelihood molecular replacement with *Phaser* (McCoy *et al.*, 2007 ▸) as implemented in the *Phenix* GUI (Liebschner *et al.*, 2019 ▸) utilizing the human IL-12B chain extracted from PDB entry 5mj3 (Desmet *et al.*, 2014 ▸) as a search model. The model was iteratively built and refined in *Coot* (Emsley *et al.*, 2010 ▸) and *BUSTER* (Blanc *et al.*, 2004 ▸) or *phenix.refine* (Adams *et al.*, 2010 ▸). The refinement strategy and progress was periodically checked with the *PDB-REDO* server (Joosten *et al.*, 2014 ▸). Riding H atoms were utilized during refinement for stricter geometry restraints and individual isotropic atomic displacement parameters were refined per atom. Some loops, in particular in the C-terminal domain, could not be unambiguously traced. The model was refined to an *R* factor of 0.229 and an *R*_free_ of 0.265. The final data-collection and refinement statistics are summarized in Table 2 ▸.

The crystallization and structure determination of the CSF-1–CSF-1R_D1–D3_ complex has previously been reported (Felix *et al.*, 2015 ▸). In brief, the data were processed in space group *I*4_1_ (unit-cell parameters *a* = 142.99, *b* = 142.99, *c* = 139.32 Å) and the structure was solved by maximum-likelihood molecular replacement (MR) in *Phaser* using the crystal structures of CSF-1 (PDB entry 3uf2; Elegheert *et al.*, 2012 ▸) and CSF-1R_D1–D3_ as found in the hIL-34–CSF-1R_D1–D3_ crystal structure (PDB entry 4dkd; Ma *et al.*, 2012 ▸). Initial refinement was performed in *BUSTER* (Blanc *et al.*, 2004 ▸) using rigid-body refinement in the first macrocycle. The final refinement steps were carried out in *Phenix* after the addition of glycan chains on Asn73, Asn45, Asn153 and Asn275 to the model. The final *R* factors reported by *Phenix* were *R* = 0.223 and *R*_free_ = 0.261; the model was further validated using *MolProbity* (Chen *et al.*, 2010 ▸).

## 3. Results   

### 3.1. Purified glycoproteins produced in GlycoDelete are reduced in size owing to glycan truncation   

**Figure 1:**
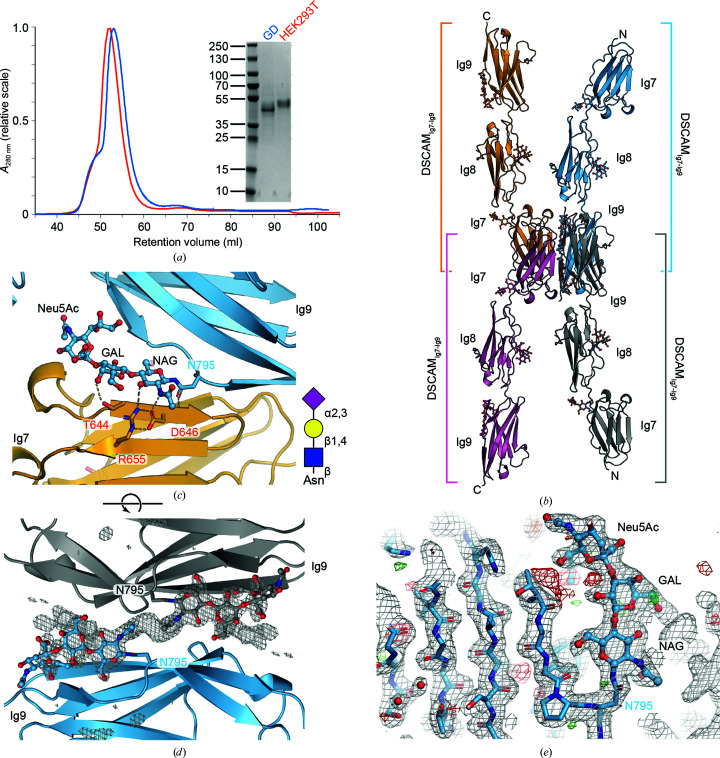
Structural analysis of DSCAM_Ig7–Ig9_ derived from HEK293 GD. (*a*) Comparison of HEK293T and HEK293 GD-derived DSCAM_Ig7–Ig9_; the inset shows reduced SDS–PAGE analysis of the purified proteins. (*b*) Cartoon representation showing four copies of DSCAM_Ig7–Ig9_ that form an oligomer by crystallographic symmetry around the glycan stub. The asymmetric unit contains a single copy. (*c*) Detailed view of the accommodation of the glycan stub at residue Asn795 in the Ig7–Ig9 interface. The inset shows a schematic representation of the glycan. (*d*) An OMIT map of the same glycan stub contoured at 3σ. (*e*) Representative electron density around the glycan at position Asn795. The 2*mF*_o_ − *DF*_c_ electron-density map is shown as a gray mesh (contoured at 1σ). Residual positive and negative *mF*_o_ − *DF*_c_ electron-density maps (contoured at ±3σ) are shown in green and red, respectively.

**Figure 2:**
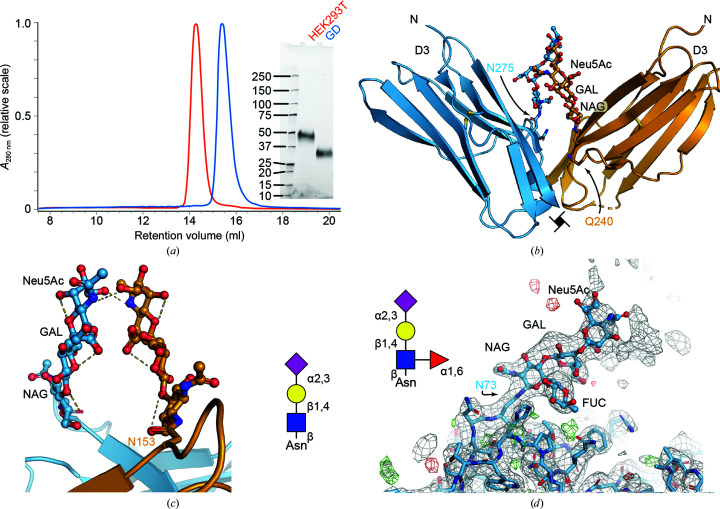
Structural analysis of CSF-1R_D1–D3_ derived from HEK293 GD. (*a*) Comparison of HEK293T and HEK293 GD-derived CSF-1R_D1–D3_; the inset shows reduced SDS–PAGE analysis of the purified proteins. (*b*) Van der Waals contacts between GD-type glycans on Asn275. (*c*) Crystal-packing contacts between symmetry-related glycans. The inset shows a schematic representation of the glycan. (*d*) The GD-type glycan on Asn73 is α-1,6-fucosylated. The 2*mF*_o_ − *DF*_c_ electron-density map is shown as a gray mesh. Residual positive and negative *mF*_o_ − *DF*_c_ electron-density maps (contoured at ±3σ) are shown in green and red, respectively.

To verify the consistent and homogeneous truncation of glycans through the use of the GlycoDelete cell line, we performed a comparative analysis of two highly glycosylated cell-surface receptor fragments and one cytokine. Expression vectors with identical protein constructs incorporated were expressed in HEK293T cells and HEK GlycoDelete cells under similar conditions. As has been observed with other engineered HEK cell lines, the protein yield for the GlycoDelete cell line is lower (approximately 60% compared with the wild type after purification). The glycoproteins produced from GlycoDelete cells consistently have a lower molecular weight as identified by SDS–PAGE (Supplementary Fig. S1). However, the shift in molecular weight is minimal for IL-12B and DSCAM, whereas for CSF-1R_D1–D3_ it entails a very substantial reduction of more than 10 kDa. To assess the level of glycosyl­ation, each sample was treated with PNGase F to identify the size of the proteins in the absence of glycosylation. IL-12B appears to contain two major glycan populations when produced in HEK293T cells, while only a single band is visible in GD-derived IL-12B. PNGase F treatment leads to an apparent single population that is approximately 5 kDa smaller (Supplementary Fig. S1). PNGase F treatment of DSCAM is efficient both for material produced in HEK293T and GD cells, leading to a further size reduction of ∼5 kDa. There is a small shift in the elution profile of DSCAM from size-exclusion chromatography, with the GD material eluting at a slightly longer retention time (Fig. 1 ▸ *a*). The shift in molecular weight as one switches from wild-type to GD HEK293S cells is most pronounced for CSF-1R_D1–D3_ (Supplementary Fig. S1 and Fig. 2 ▸ *a*). Comparison of the size-exclusion chromatography profiles of CSF-1R_D1–D3_ derived from HEK293T and HEK293 GlycoDelete cells on an analytical Superdex 200 Increase 10/300 GL column showed that CSF-1R_D1–D3_ of GD origin elutes 1.5 ml later (with a total bed volume of 24 ml), indicating a remarkably longer retention time on the column (Fig. 2 ▸ *a*). This decrease in molecular weight was confirmed by SDS–PAGE gel analysis, where the GD-derived CSF-1R_D1–D3_ runs 10 kDa lower than CSF-1R_D1–D3_ with wild-type glycans and runs only slightly higher than fully deglycosylated CSF-1R_D1–D3,_ indicating a decreased size and increased homogeneity that are entirely attributed to the change in glycosylation (Fig. 2 ▸ *a*). Both the CSF-1R and DSCAM GlycoDelete materials show lower molecular-weight bands on the SDS–PAGE. This possibly indicates that the truncation of the glycans makes the proteins more sensitive to degradation, because the glycans cover a smaller area of the protein surface. Clearly, the processing of *N*-glycosylation in the GlycoDelete cell line has an effect on the overall size and homogeneity of the glycoproteins.

### 3.2. GlycoDelete facilitates crystal structure determination of the netrin-binding portion of DSCAM_Ig7–Ig9_   

Down syndrome cell-adhesion molecule (DSCAM) is a cell-surface glycoprotein with an extensive ectodomain consisting of ten immunoglobulin domains and six fibronectin domains. The DSCAM gene is situated on chromosome 21 and is overexpressed in Down syndrome patients (Yamakawa *et al.*, 1998 ▸). DSCAM has been implicated in congenital heart disease (Grossman *et al.*, 2011 ▸), and in *Drosophila* DSCAM is involved in axon guidance and neuronal wiring (Schmucker *et al.*, 2000 ▸; Meijers *et al.*, 2007 ▸). DSCAM interacts with several guidance molecules, notably netrin (Ly *et al.*, 2008 ▸). To further characterize the portion of the ectodomain of human DSCAM that interacts with netrin, we produced a truncated fragment consisting of immunoglobulin domains Ig7, Ig8 and Ig9. There are five putative *N*-glycosylation sites in this fragment according to the *NetNGlyc* 1.0 server (Blom *et al.*, 2004 ▸).

Purified DSCAM_Ig7–Ig9_ protein derived both from wild-type and GD cells was concentrated to 5–7.5 mg ml^−1^ and was submitted to crystallization using the same set of crystallization screens. Crystals were obtained from both sources using similar mother-liquor formulations from related conditions that are present in The PEGs II Suite from Qiagen (see Table 1 ▸). Wild-type-derived rod-shaped crystals were obtained of significant size and good morphology, but X-ray diffraction screening on the microfocus beamline P14 only resulted in diffraction patterns to ∼8 Å resolution. GD-derived crystals from the initial condition diffracted to 2.5 Å resolution and further optimization of the conditions resulted in a complete data set from a single crystal at 1.85 Å resolution (Table 2 ▸).

The crystal structure of human DSCAM_Ig7–Ig9_ contains one molecule in the asymmetric unit. The residues Val595–Glu884 are visible in the electron density of the refined structure. One-residue cloning scars were observed at both of the protein termini (Thr594 and Glu885, respectively). Only the C-terminal 6×His tag was not visible. The fragment corresponds to three immunoglobulin (Ig)-type C2 domains, each consisting of two layers of antiparallel β-sheets held together by a conserved disulfide bond. Clear electron density for the glycan chains was observed in all three Ig domains at residues Asn666, Asn710, Asn748 and Asn795. The predicted glycan at Asn658 was not observed in the electron density. For three solvent-exposed glycosylation sites, the glycan stub is only partially visible in the electron density. Interestingly, the glycan stub attached to Asn795 within the Ig9 domain shows the full *N*-glycosylation pattern (Neu_5_AcGalGlcNAc) of the HEK293 GlycoDelete cell line (Figs. 1 ▸ *c*, 1 ▸ *d* and 1 ▸ *e*). This glycan plays a key role in the assembly of a crystallographic tetramer formed by the interaction of two Ig9 and two Ig7 domains (Fig. 1 ▸ *b*). The glycan stub sits at the center of this oligomer and makes contacts with the symmetry-related glycan stub from the other Ig9 molecule that is part of the interface (Figs. 1 ▸ *b* and 1 ▸ *c*). The stub occupies a central portion of the Ig7–Ig9 interface (Figs. 1 ▸ *b*,  ▸1*c* and 1 ▸ *d*), making hydrogen bonds between the glycan units and protein residues from neighboring DSCAM_Ig7–Ig9_ molecules.

This raises the question whether the truncated glycan stub may create artificial protein–protein interfaces. The most critical question that the human DSCAM_Ig7–Ig9_ structure could answer is whether the dimer interface that has been observed in the *Drosophila* DSCAM Ig7 domain is evolutionarily conserved (Li *et al.*, 2016 ▸; Sawaya *et al.*, 2008 ▸). An analysis of the buried surface areas of all of the contacts occurring in the crystal lattice reveals that the most extensive protein interface is formed between Ig7 and a symmetry-related Ig7 domain (830 Å^2^). When this protein interface is compared with the Ig7–Ig7 interface observed in available *Drosophila* DSCAM crystal structures, it is remarkably similar (Supplementary Fig. S2). This indicates that the glycan stub-mediated crystal contacts have not disrupted the functional oligomerization of the DSCAM Ig7–Ig9 fragment in the crystal lattice. Within the Ig9–Ig7–Ig7–Ig9 tetramer interface, the protein interfaces contribute a buried surface area of 1397 Å^2^ (Ig9–Ig9 = 349 + Ig9–Ig7 = 218 + Ig7–Ig7 = 830 Å^2^). The glycan stub extending from Ig9 that mediates the crystal contacts contributes an additional 345 Å^2^, which is more than the Ig9–Ig7 protein interface alone. We conclude therefore that the rigidity of the glycan stub substantially contributes to crystal formation, but without disrupting the biologically relevant dimerization of the receptor fragment.

### 3.3. GlycoDelete glycan stubs contribute to the crystal lattice in CSF-1–CSF-1R_D1–D3_ crystals   

As a member of the class III receptor tyrosine kinases, colony-stimulating factor 1 receptor (CSF-1R) consists of an ectodomain comprising five Ig-like domains, a transmembrane helix and an intracellular kinase domain. Activation occurs through the binding of CSF-1 or IL-34 to the second (D2) and third (D3) Ig domains in the CSF-1R ectodomain, eliciting ligand-induced dimerization and initiation of the intracellular signaling cascade (Verstraete & Savvides, 2012 ▸). CSF-1R signaling is required for survival, differentiation and proliferation in cells of the mononuclear phagocytic cell lineage such as macrophages, monocytes and placental trophoblasts and cells of the central nervous system such as microglia cells (Stanley & Chitu, 2014 ▸).

In order to obtain well diffracting crystals, expression in glycan-engineered HEK cells was combined with the introduction of point mutations at each of the confirmed N-linked glycosylation sites (Asn73, Asn153, Asn240 and Asn275), mutating the *N*-glycosylated asparagine to glutamine. Only three of the mutants proved to be amenable for large-scale production, as initial expression tests showed that abolishing the Asn73 glycosylation site resulted in loss of expression. In the first instance, we expressed the glycosylation mutants in HEK293T and HEK293S *MGAT1^−/−^* cells. Crystals of the receptor expressed in HEK293T cells looked like spherulites and did not diffract, while crystals containing the receptor with HEK293S *MGAT1^−/−^* glycans had a rectangular appearance and diffracted to a high-resolution limit of 4.5 Å; they could be indexed in space group *I*4_1_, with unit-cell parameters *a* = *b* = 143.0, *c* = 139.2 Å. In order to further improve the X-ray diffraction quality of the crystals, we attempted to trim the glycans of the protein produced in HEK293S *MGAT1^−/−^* to the first GlcNAc residue by EndoH digestion. However, truncation of all of the remaining glycans resulted in a pronounced reduction in solubility and crystal screens could only be set up at 4 mg ml^−1^, albeit without yielding crystallization hits. A less drastic truncation of the N-linked glycans could be achieved by expressing the CSF-1R_D1–D3_ glycosylation mutants in the GlycoDelete cell line. Each of the CSF-1R_D1–D3_ constructs was transfected in GlycoDelete cells according to the same protocol for HEK293T and HEK293S *MGAT1^−/−^* cells. The final yield of purified CSF-1R_D1–D3_ was reduced to about 2 mg per litre of culture, which is still enough to allow extensive screening of crystallization conditions.

Despite the decrease in glycan size, the CSF-1–CSF-1R_D1–D3_ complex retained a solubility close to that of the wild-type material and could be concentrated to 7.4 mg ml^−1^. After extensive screening, crystals appeared in related conditions from the Crystal Screen Lite and Index screens. Data collection on the ID23-1 beamline at the ESRF synchrotron resulted in a data set with a high-resolution limit of 2.8 Å. These high-resolution data allowed us to rationalize the impact of the glycan engineering on crystal lattice formation. Crystals from HEK293S *MGAT1^−/−^* cells have a similar packing to those obtained from GlycoDelete, based on the similarity in space group and unit-cell parameters. This indicates that protein–protein interactions dominated the crystal lattice formation, but the reduction in glycan structure improved the crystal order, leading to better diffraction properties, as observed for the DSCAM_Ig7–Ig9_ crystals. The N240Q point mutation was crucial to allow crystal-packing contacts between symmetry-related D3 domains (Fig. 2 ▸ *b*). The side chain of Gln240 points towards the D3 packing interface, leaving no space to accommodate an N-linked glycan. Additionally, the same interface shows a contribution of the GD-type glycan at Asn275 to lattice formation by van der Waals contacts with its symmetry-related counterparts (Fig. 2 ▸ *b*). Such GD glycan-mediated packing interactions were also observed for domain 1, where the *N*-acetylneuraminic acid groups of two Asn153-linked glycans form hydrogen bonds (Fig. 2 ▸ *c*, Supplementary Fig. S3). Finally, density for an α-1,6-linked fucose was observed on the Asn73 glycan, confirming that this is a possible modification for a GD-type glycan.

### 3.4. GlycoDelete cells do not alter the structural glycan on IL-12B but reduce the size and heterogeneity of the other glycans   

IL-12B is a soluble cytokine receptor-like protein associated with the heterodimeric pro-inflammatory cytokines interleukin-12 (IL-12) and interleukin-23 (IL-23). IL-12B is secreted as a monomer (denoted IL-12p40) and as a homodimer (denoted IL-12p80). IL12B can also form a disulfide-linked interaction mediated by Cys197 on murine IL-12p40 to form heterodimeric cytokines with the IL-12p35 subunit, resulting in IL-12, or the IL-23p19 subunit, resulting in IL-23. The IL-12p40 and IL-12p80 oligomers are competitive inhibitors of IL-12 and IL-23 signaling as they can bind to the IL-12Rβ1 receptor, which is the low-affinity receptor for these cytokines. The IL-12p80 homodimer has been attributed with pleiotropic agonistic effects ranging from modulating the chemotactic behavior of macrophages and dendritic cells to triggering lymphotoxin-α production in microglia (Cooper & Khader, 2007 ▸); however, more research will be necessary for a more complete functional annotation of this molecular species.

**Figure 3:**
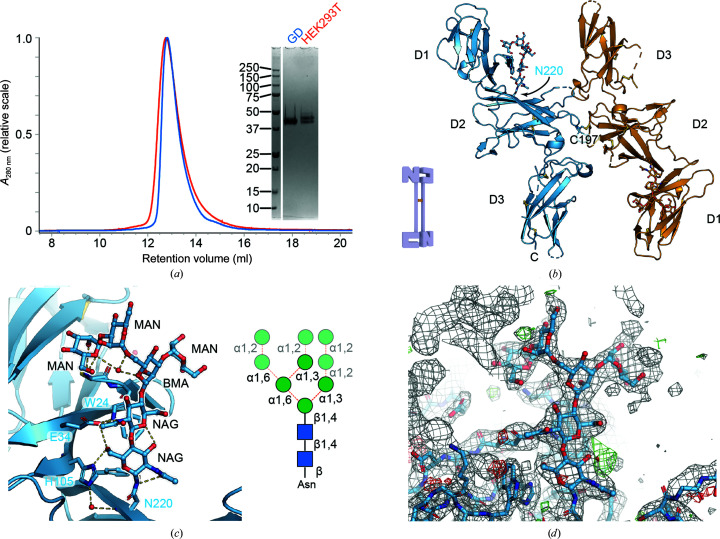
Structural analysis of IL-12B derived from HEK293 GD. (*a*) Comparison of HEK293T and HEK293 GD-derived IL-12B; the inset shows SDS–PAGE analysis of the purified proteins under reducing conditions. (*b*) Cartoon representation of the IL-12B homodimer formed through crystallographic symmetry (a twofold crystallographic axis is present at Cys197). (*c*) Close-up of the immature N-linked glycan present on Asn220 and its extensive interactions with the neighboring protein. The inset shows a schematic representation of the glycan; the mannose residues that could not be readily identified in the electron density are partially transparent. (*d*) Representative electron density around the glycan at position Asn220. Density at the top left belongs to a symmetry mate. The 2*mF*_o_ − *DF*_c_ electron-density map is shown as a gray mesh. Residual positive and negative *mF*_o_ − *DF*_c_ electron-density maps (contoured at ±3σ) are shown in green and red, respectively.

The crystal structure of murine IL-12B presented here (Fig. 3 ▸ *b*) displays high overall structural similarity to human IL-12B (r.m.s.d. of 0.85 Å over 224 C^α^ atoms), which would be expected from the 66% sequence identity between them. The IL-12p40 protomer adopts an S-like conformation, with the Ig-like D1 sitting, out of plane, at a near-perpendicular angle to D2, followed by D3, in plane, again at a near-perpendicular angle (Fig. 3 ▸ *b*). The fibronectin domains D2 and D3 form what is known as the cytokine-binding homology region (CHR). The CHR should contain a WS*X*WS motif, which is a hallmark of type 1 cytokine receptors, in the C-terminal domain (D3). A degenerate form of this motif is present in the segment 323-Cys-Ser-Lys-Trp-Ala-327. Unlike the canonical motif, this sequence is not amenable to *C*-mannosylation and does not engage in an extensive zipper-like arrangement. The crystallographic twofold axis present in the *I*4_1_ lattice is located at Cys197, thereby forming a crystallographic homodimer. Aside from the homotypical disulfide, the interface area is very limited in size (287 Å^2^) and lacks specific interactions (13 interface residues per protomer and no hydrogen bonds or salt bridges).

Only the N-linked glycan at Asn220 is clearly present (Fig. 3 ▸ *c*), while other glycosylation sites are likely to be flexible or less occupied, as suggested by the electrophoretic mobility of the recombinant protein (Supplementary Fig. S1). Mouse IL-12B was reported to contain a peculiar *N*-glycosylation pattern (Bootz *et al.*, 2016 ▸), and this glycan is of the immature type and not a GlycoDelete trisaccharide (Figs. 3 ▸ *c* and 3 ▸ *d*). Extensive van der Waals contacts are present between Trp24 and the nonterminal NAG sugar as well as hydrogen bonds between Met23, Glu34 and His105 and other parts of the glycan (Fig. 3 ▸ *c*). The electron density allows the modeling of a Man_4_GlcNAc_2_ glycan. Previous research and DNA sequencer-aided fluorophore-assisted carbohydrate electrophoresis analysis identified this glycan to be Man_9_GlcNAc_2_ or Man_8_GlcNAc_2_ (Fig. 3 ▸ *d*). Thus, it appears that the immature glycan is needed for the proper functioning of IL-12B, probably to ensure proper folding and stability. Although the GD cell line was engineered to alter the glycosylation machinery, it does not affect the formation and retention of the immature glycan. This immature glycan is probably formed and partially shielded from the solvent prior to reprocessing by the EndoT enzyme. The immature glycan is located between the D1 and D2 domains and may be difficult to access by the EndoT enzyme. We conclude that it is a critical feature of the GD cell line that the glycosylation is only reduced after it passes into the Golgi, as it retains the capability to secrete glycoproteins that require immature glycans for folding.

## 4. Discussion   

The heterogeneity in *N*-glycosylation (NG) has been a longstanding issue in structural biology (Chang *et al.*, 2007 ▸) and is commonly addressed at three different time points during protein expression and purification. (i) The NG motif (Asn-**Pro**-Ser/Thr) is disrupted by mutagenesis during construct design. (ii) The choice of expression host takes into consideration the capability of the host glycosylation machinery to produce homogeneous glycans. (iii) After protein expression, the purified protein can be enzymatically treated with recombinantly produced endoglycosidases or exoglycosidases to truncate all of the glycans present into stubs.

To disrupt the NG motif, the asparagine residue is often mutated to a glutamine, which is no longer a substrate for the oligosaccharyltransferase. Other less conservative substitutions of the asparagine, or targeting the Ser/Thr site that is part of the NG motif, will also disrupt the glycosylation of a specific site. However, not all NG sites can be mutated, as some glycosylation sites are crucial for proper protein folding and stability. Careful exploration of mutants is advisable (Felix *et al.*, 2015 ▸; Verstraete *et al.*, 2017 ▸, 2014 ▸), but can become a prohibitively daunting undertaking when many NGs are present on the protein(s) of interest. It is of course possible to obtain diffraction-quality crystals from glycoproteins and their complexes with wild-type HEK293 cells (Felix *et al.*, 2016 ▸; Finci *et al.*, 2014 ▸; Liu *et al.*, 2018 ▸), but for many glycoproteins crystallization is reported but the structure is never determined (see, for example, Kulahin *et al.*, 2004 ▸). As illustrated by the case study on DSCAM_Ig7–Ig9_, it is possible to obtain well proportioned crystals from wild-type HEK cells that unfortunately do not diffract X-rays well. Using the modified GD cell line led to the production of DSCAM_Ig7–Ig9_ protein that is essentially identical in composition to the wild-type material, except for a reduction in the complexity of the five glycans that are present. The crystallization conditions for wild-type and GD-derived DSCAM_Ig7–Ig9_ are almost identical (Table 1 ▸), yet a crystal structure could only be determined with the GD-derived DSCAM_Ig7–Ig9_ crystals.

Alternative strategies that facilitate the production of target proteins with less complex and enzyme-treatable *N*-glycosyl­ation are often followed (Aricescu & Owens, 2013 ▸; Büssow, 2015 ▸). The choice of expression host strongly affects the size, complexity and homogeneity of *N*-glycosylation. Insect cell lines produce paucimanosidic NG, yeast cell lines produce high-mannose NG and mammalian cell lines tend to produce a complex mix of NG (Van Landuyt *et al.*, 2019 ▸). Especially for structural studies on human and other mammalian proteins, mammalian cell lines can be employed that are engineered to modify the enzymatic production of N-glycans. A highly effective enzyme to target in the glycosylation pathway is *N*-acetylglucosaminyltransferase I (MGATI/GnTI). Without this enzyme, the glycosylation pathway stalls at the Man_5_GlcNac_2_ stage. These glycans are no longer recognized by certain lectins such as ricin. The CHO Lec3.2.8.1 (Chaney *et al.*, 1989 ▸) and HEK293S *MGAT1^−/−^* (Reeves *et al.*, 2002 ▸) cell lines lack N-glycans of high complexity and have been successfully used in crystallization efforts. While this cell line has yielded many glycoprotein crystals with good diffraction properties, further reduction of the glycan complexity could be beneficial for structural characterization by X-ray crystallography.

In addition to cell-line engineering, the *N*-glycosylation pathway can also be manipulated by the addition of *N*-glycosylation processing inhibitors during protein expression. Two well known inhibitors are kifunensine and swainsonine, which halt *N*-glycosylation by inhibiting ER-resident α-mannosidase I and Golgi-resident α-mannosidase II, respectively (Elbein *et al.*, 1982 ▸, 1990 ▸). These inhibitors lead to more homogeneous glycans of the high-mannose type. Kifunensine is more used than swainsonine since it is active at lower concentrations. Glycoproteins produced in kifunensine-treated HEK293T cells can be directly amenable to crystallization (Felix *et al.*, 2015 ▸), although more often the glycans are further trimmed by endoglycosidase digests. To further reduce the size and heterogeneity of NG, enzymatic treatment with endoglycosidases and exoglycosidases can be applied. Jack-bean mannosidase can be used to trim high-mannose glycans to a trisaccharide Man-GlcNAc_2_ core (Bloch *et al.*, 2018 ▸). More commonly, endoglycosidase H (EndoH) is used, since it cleaves high-mannose-type NG glycans within the GlcNAc–GlcNAc core to leave a single GlcNAc unit attached to the asparagine (Imperiali & O’Connor, 1999 ▸). Such high-mannose-type glycans are for example produced by the aforementioned HEK293S *MGAT1^−/−^* cell line, and protein expression in *MGAT1^−/−^* cells is often combined with EndoH treatment. This has the advantage that the preservation of a single mannose unit maintains the solubility of the glycoprotein. However, this two-step procedure can drastically reduce the protein yield, and may still result in aggregation after enzymatic treatment. This is nicely illustrated by the case study presented here on the CSF-1R fragment, which aggregated after EndoH treatment. The GD cell line has the advantage that the reduction in glycan size and its complexity occurs within the cell during protein expression, and if the glycoprotein is secreted in sufficient quantities no further processing of the glycans is required.

It is important to note that the general glycosylation machinery is not affected in the GD cell line, despite the removal of the *N*-acetylglucosaminyltransferase I and the redirection of EndoT to the Golgi. This is illustrated by the case study on IL-12B, which requires an immature glycan for proper folding. Aside from the more diverse complex and hybrid-type glycans, immature ER-type or high-mannose-type glycans are common in the mammalian proteome (Riley *et al.*, 2019 ▸) and on viral spike proteins (Watanabe *et al.*, 2020 ▸). Solvent accessibility to the N-linked glycosylation site in the context of the folded protein is a key factor determining the potential maturation of the glycan (Lee *et al.*, 2014 ▸). The immature glycan was clearly present in the crystal structure of IL-12B based on recombinant protein produced by GD cells, illustrating the general versatility of this cell line for structural biology applications.

## Supplementary Material

PDB reference: colony-stimulating factor 1 receptor, 4wrl

PDB reference: Down syndrome cell-adhesion molecule, 6zr7

PDB reference: murine IL-12B, 6sff

Supplementary Figures. DOI: 10.1107/S2059798320013753/wa5128sup1.pdf

## Figures and Tables

**Table 1:** Crystallization conditions for DSCAM_Ig7–Ig9_ for the wild-type and GD-derived proteins

	HEK293T	HEK293 GlycoDelete
Protein sample	5 mg ml^−1^ in 20 m*M* HEPES pH 7.4, 100 m*M* NaCl, 1 m*M* DTT	7.5 mg ml^−1^ in 20 m*M* HEPES pH 7.4, 100 m*M* NaCl, 1 m*M* DTT
		
Initial crystal	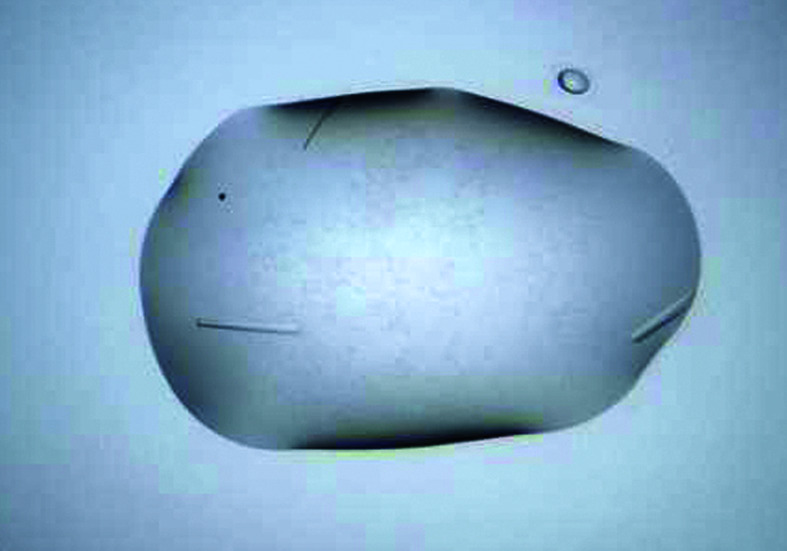	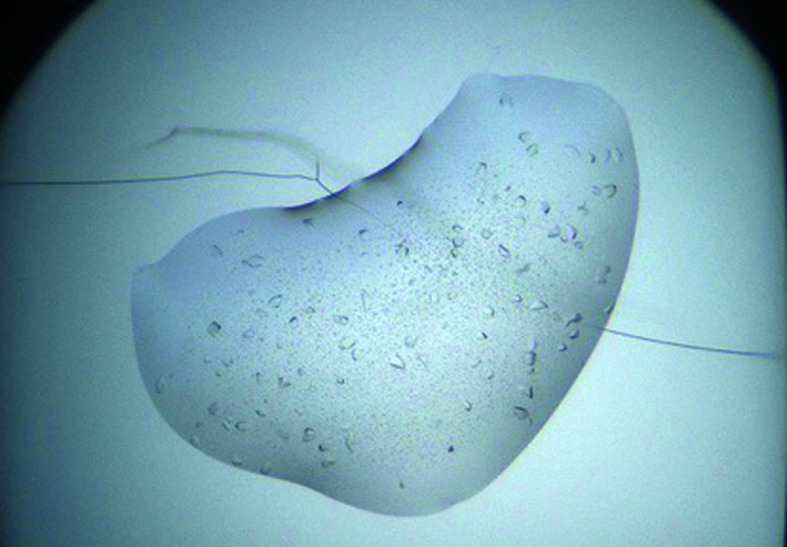
Screen	The PEGs II Suite	The PEGs II Suite
Condition	H9: 0.05 *M* magnesium acetate, 10%(*w*/*v*) PEG 8000, 0.1 *M* sodium acetate	H8: 0.2 *M* calcium acetate, 10%(*w*/*v*) PEG 8000. 0.1 *M* HEPES pH 7.5
Protein sample	7 mg ml^−1^ in 20 m*M* HEPES pH 7.4, 100 m*M* NaCl, 1 m*M* DTT	7 mg ml^−1^ in 20 m*M* HEPES pH 7.4, 100 m*M* NaCl, 1 m*M* DTT
Optimized condition	0.05 *M* magnesium acetate, 16%(*w*/*v*) PEG 8000	0.2 *M* calcium acetate, 10%(*w*/*v*) PEG 8000, 0.1 *M* HEPES pH 7.5, 3%(*v*/*v*) glycerol
		
Optimized crystal mounted in a cryo-loop	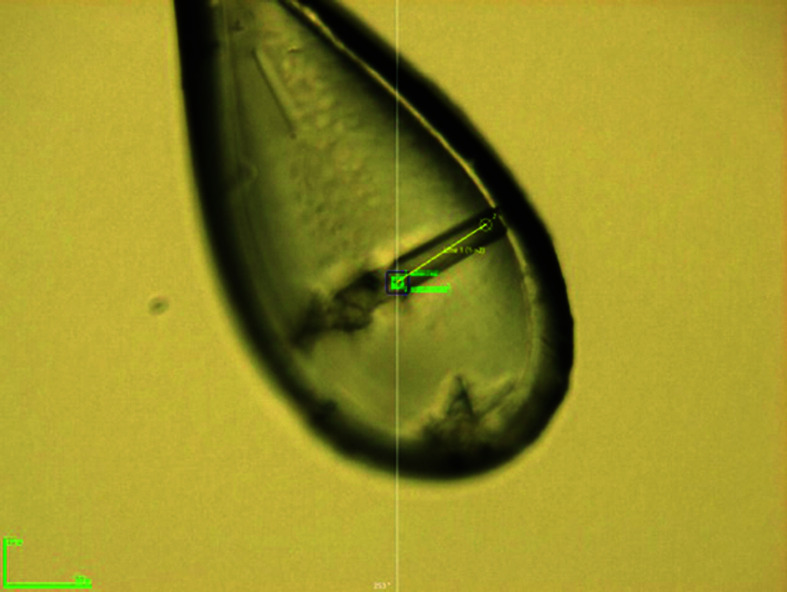	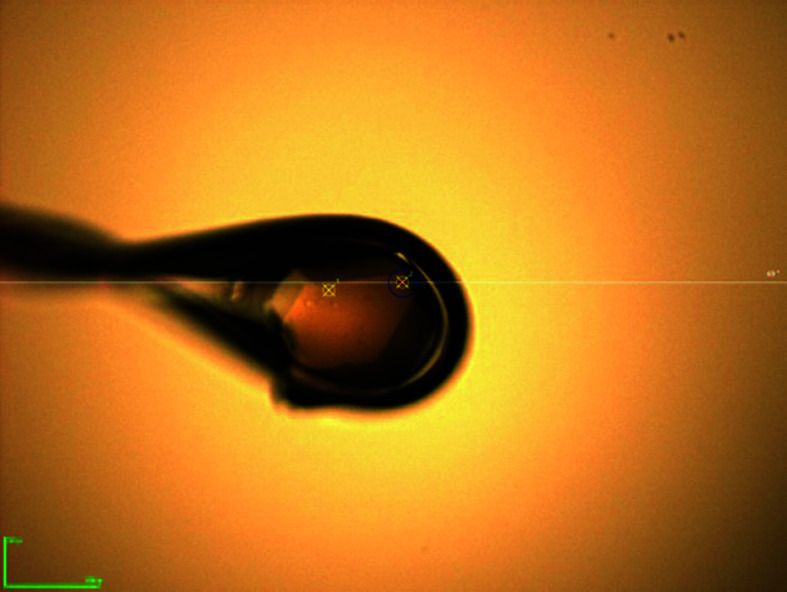
Diffraction pattern	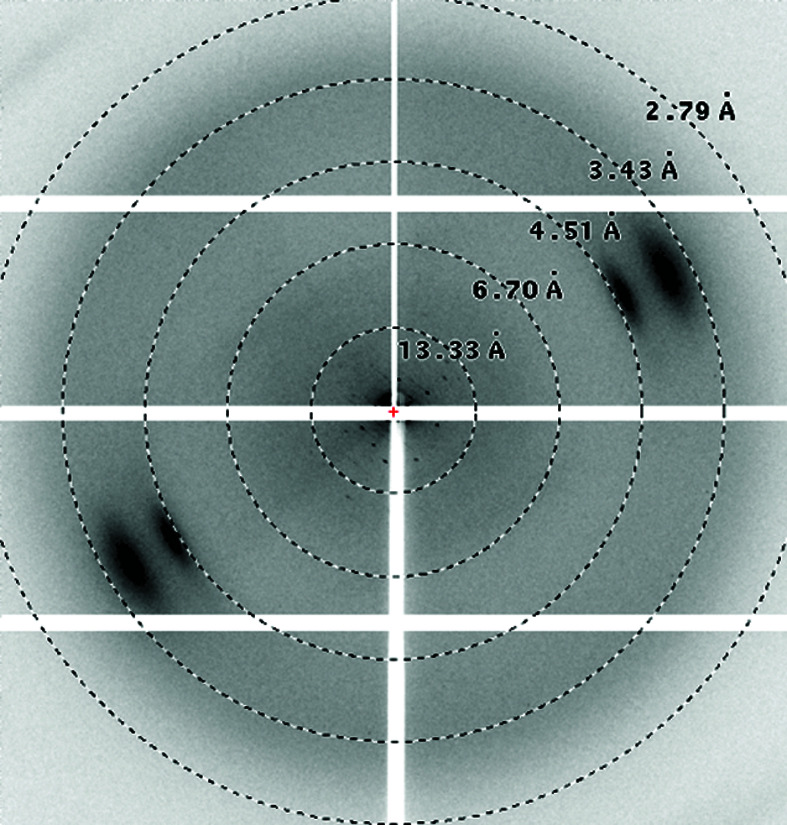	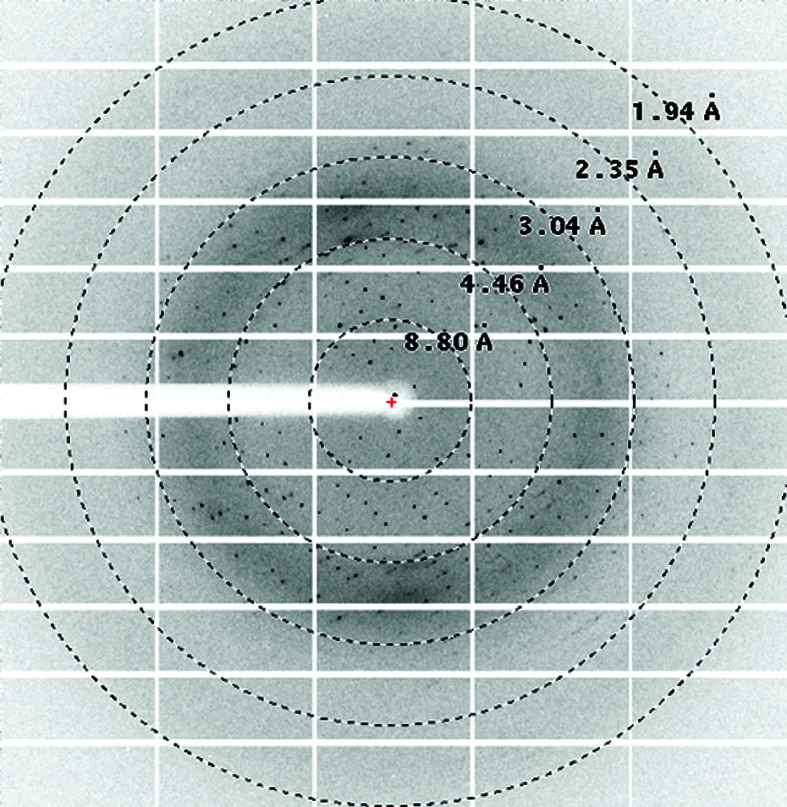
Diffraction limit (Å)	∼8	1.85

**Table 2:** Crystallographic data-collection and refinement statistics Values in parentheses are for the highest resolution shell.

	DSCAM_Ig7–Ig9_	IL-12B	CSF-1–CSF-1R_D1–D3_
Wavelength (Å)	0.976	1.033	0.976
Resolution range (Å)	84.81–1.85 (1.89–1.85)	67.41–2.40 (2.49–2.40)	49.71–2.80 (2.97–2.80)
Space group	*C*2	*I*4_1_	*I*4_1_
*a*, *b*, *c* (Å)	78.6, 71.4, 92.2	85.88, 85.88, 107.67	143.00, 143.00, 138.32
α, β, γ (°)	90, 113.1, 90	90, 90, 90	90, 90, 90
Total reflections	148955 (9215)	123592 (9760)	235463 (37046)
Unique reflections	39662 (2418)	15233 (1486)	34125 (5448)
Multiplicity	3.8 (3.8)	8.11 (6.6)	6.9 (6.8)
Completeness (%)	99.0 (97.4)	99.7 (97.4)	99.8 (99.2)
Mean *I*/σ(*I*)	12.8 (1.6)	12.2 (0.8)	20.7 (2.3)
Wilson *B* factor (Å^2^)	43.1	60.25	82.3
*R*_merge_	0.044 (0.874)	0.117 (1.564)	0.059 (0.864)
*R*_meas_	0.051 (1.011)	0.125 (1.698)	0.064 (0.815)
*R*_p.i.m._	0.026 (0.502)	0.044 (0.649)	0.024 (0.351)
CC_1/2_	1.0 (0.7)	0.998 (0.487)	0.999 (0.74)
Reflections used in refinement	37658 (2753)	15215 (1480)	34116 (3318)
Reflections used for *R*_free_	2001 (153)	1511 (144)	1706 (165)
*R*_work_	0.189 (0.289)	0.229 (0.365)	0.223 (0.409)
*R*_free_	0.217 (0.303)	0.265(0.437)	0.261 (0.465)
No. of non-H atoms			
Total	2564	2412	6657
Macromolecules	2281	2306	6370
Ligands	116	73	287
Solvent	167	33	n.a.
No. of protein residues	292	295	833
R.m.s.d., bond lengths (Å)	0.01	0.005	0.006
R.m.s.d., angles (°)	1.80	1.04	1.094
Ramachandran favored (%)	96.9	95.79	95.6
Ramachandran allowed (%)	3.1	4.21	4.4
Ramachandran outliers (%)	0.0	0.00	0.0
Average *B* factor (Å^2^)			
Overall	36.9	66.8	100.3
Macromolecules	33.2	66.4	97.4
Ligands	74.8	85.7	164.6
Solvent	60.4	56.0	n.a.
No. of TLS groups	4	n.a.	18
PDB code	6zr7	6sff	4wrl
