# Supplementary material for: Homogeneously N-glycosylated proteins derived from the GlycoDelete HEK293 cell line enable diffraction-quality crystallogenesis
Source: Acta Crystallogr D Struct Biol. 2020 Nov 24;76(Pt 12):1244–55. doi: 10.1107/S2059798320013753 (PMC7709199; doi:10.1107/S2059798320013753)
Supplement: Supplementary file 1 [file d-76-01244-sup1.pdf]

Acta Cryst D STRUCTURAL BIOLOGY

Volume 76 (2020)

Supporting information for article:

Homogeneously *N*-glycosylated proteins derived from the GlycoDelete HEK293S cell line enable diffraction-quality crystallogenesis

Sandra Kozak, Yehudi Bloch, Steven De Munck, Aleksandra Mikula, Isabel Bento, Savvas N. Savvides and Rob Meijers

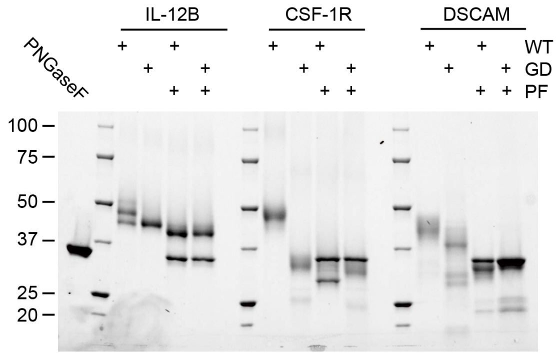

Figure S1 Comparison of electrophoretic mobilities of glycosylated IL-12B, CSF-1R and $DSCAM_{Ig7\text{-}Ig9}$ produced in WT and GD HEK293 cell lines. Shown is a Coomassie-stained SDS-PAGE analysis of the recombinant proteins under reducing conditions including samples that were treated with PNGaseF. Wild-type HEK293 (WT), HEK293 GlycoDelete (GD), PNGaseF (PF).

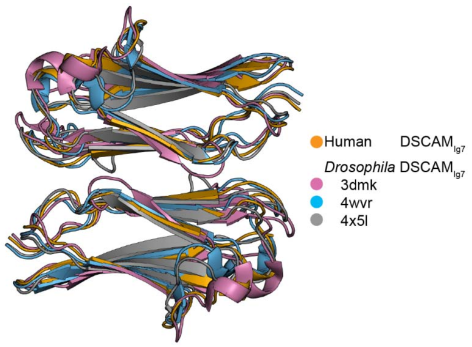

Figure S2 Ig7/Ig7 dimer interface observed in human and Drosophila Dscam structures. Superimposed structures of human DSCAM Ig7 (orange) and Drosophila Dscam Ig7 (3dmk in pink, 4wvr in blue and 4x5l in grey) dimers. Strucutres aligned to human DSCAM 7 as reference, the R.M.S.D. values for 196 Ca atoms are 1.279,2.154 and 2.095 A for the 4wvr,4x5l and 3dmk structures respectively.

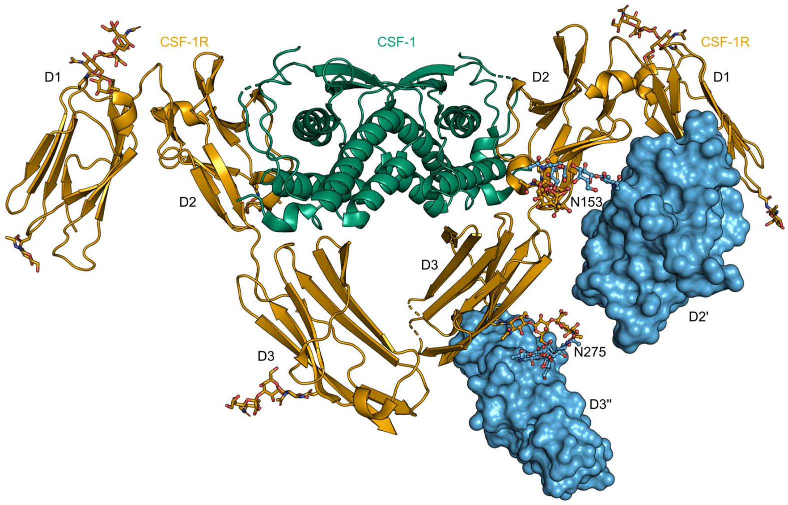

Figure S3 Glycan mediated crystal lattice contacts mapped on the complete asymmetric unit of CSF-1:CSF-1R$_{D1\text{-}D3}$. Asymmetric unit containing a CSF-1 homodimer (green) and two CSF-1R $_{D1\text{-}D3}$ chains (orange) with two domains from symmetry related CSF-1R $_{D1\text{-}D3}$ (Blue surface) forming glycan mediated crystal packing contacts using either N153 (D2') or N275 (D3'').
